# How Are Synapses Born? A Functional and Molecular View of the Role of the Wnt Signaling Pathway

**DOI:** 10.3390/ijms24010708

**Published:** 2022-12-31

**Authors:** Christian Bonansco, Waldo Cerpa, Nibaldo C. Inestrosa

**Affiliations:** 1 Centro de Neurobiología y Fisiopatología Integrativa (CENFI), Instituto de Fisiología, Facultad de Ciencias, Universidad de Valparaíso, Valparaíso 2360102, Chile; 2 Laboratorio de Función y Patología Neuronal, Departamento de Biología Celular y Molecular, Facultad de Ciencias Biológicas, Pontificia Universidad Católica de Chile, Santiago 8331150, Chile; 3 Centro de Excelencia en Biomedicina de Magallanes (CEBIMA), Universidad de Magallanes, Punta Arenas 6200000, Chile; 4 Centro de Envejecimiento y Regeneración (CARE UC), Departamento de Biología Celular y Molecular, Facultad de Ciencias Biológicas, Pontificia Universidad Católica de Chile, Santiago 8331150, Chile

**Keywords:** immature synapses, synaptic conversion, developmental plasticity, GluN2B-containing glutamate receptors

## Abstract

Synaptic transmission is a dynamic process that requires precise regulation. Early in life, we must be able to forge appropriate connections (add and remove) to control our behavior. Neurons must recognize appropriate targets, and external soluble factors that activate specific signaling cascades provide the regulation needed to achieve this goal. Wnt signaling has been implicated in several forms of synaptic plasticity, including functional and structural changes associated with brain development. The analysis of synapses from an electrophysiological perspective allows us to characterize the functional role of cellular signaling pathways involved in brain development. The application of quantal theory to principles of developmental plasticity offers the possibility of dissecting the function of structural changes associated with the birth of new synapses as well as the maturation of immature silent synapses. Here, we focus on electrophysiological and molecular evidence that the Wnt signaling pathway regulates glutamatergic synaptic transmission, specifically N-methyl-d-aspartate receptors (NMDARs), to control the birth of new synapses. We also focus on the role of Wnts in the conversion of silent synapses into functional synapses.

## 1. Introduction

During the development of the central nervous system (CNS), each synaptic contact must undergo a series of events that involve plastic changes in both pre- and post-synaptic elements [1]. Pre- and post-synaptic membranes require the interplay of signals that regulate the synthesis machinery, release neurotransmitters, and the trafficking of receptors [2]. Moreover, the active milieu concept includes presynaptic-glia-postsynaptic communication through a varied set of gliotransmitters and neuroactive factors that promote the development, maturation, and modulation of synaptic transmission [3]. Although the advent of new microscopic techniques (i.e., confocal, multiphoton, and super-resolution microscopy) has furthered knowledge of the structural and molecular basis of synaptic maturation, the electrophysiological approach must be employed to establish the functional correlate [4,5,6]. Here, we evaluate current findings implicating Wnt pathway activation as a possible signal in the birth and conversion of silent synapses.

Based on electrophysiological and molecular findings, we propose that Wnt ligands may regulate glutamatergic synaptic transmission through the activation of the mobilization of GluN2B-containing N-methyl-D-aspartate (NMDA) receptors in the post-synaptic membrane of nascent synapses and through the subsequent insertion of the α-amino-3-hydroxy-5-methyl-4-isoxazolepropionic acid (AMPA) receptors that are required for the complete conversion of silent synapses.

### Development of a Silent Synapse

**Figure 1:**
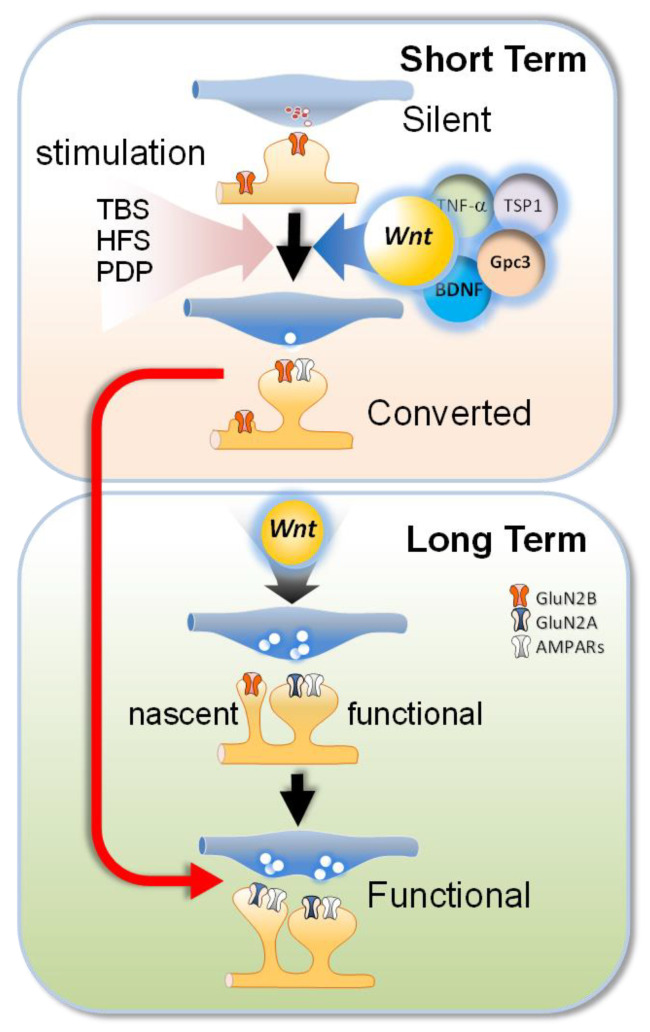
Synaptic formation and functional maturation of glutamatergic synapses. During synaptogenesis, Wnt agonists may thereby induce the birth of dendritic spines, promoting the trafficking of GluN2B-containing receptors to nascent synapses. The transformation of silent synapses (i.e., solely express GluN2B) into converted synapses can be triggered by several neuritogenic factors, including Wnt, Brain-derived neurotrophic factor (BDNF), Tumor necrosis factor α (TNFα), glypicans (Gpc), and astrocyte-secreted thrombospondins (TSP). In converted synapses, Wnt agonists could promote the developmental switch, changing GluN2B-containing receptors to GluN2A-containing receptors. Neuronal activity as in several plasticity induction protocols (i.e., paired-depolarization protocol, PDP; theta burst stimulation, TBS; high-frequency stimulation, HFS) induces AMPAR insertion completing the maturation of functional synapses endowed with GluN2A-containing NMDARs and AMPARs. The activation of the Wnt and BDNF pathways in presynaptic terminals is involved in the coordinated maturation of the presynaptic machinery and post-synaptic elements.

The existence of ineffective synapses and their functional conversion as a major strategy in the developmental plasticity of glutamatergic hippocampal synapses was first proposed by Malinow and Malenka [7,8]. Using the minimal-stimulation technique to putatively activate a single synapse [9], these pioneering studies described silent synapses in CA1 pyramidal neurons. In these silent synapses, synaptic stimulation evoked NMDAR-mediated post-synaptic currents only at depolarized potentials; no responses occurred at physiological values of resting membrane potential. The explanation that was initially proposed for this phenomenon suggested that the ineffectiveness of the silent synapses is due to a lack of AMPARs; only NMDAR expression is observed at such synapses, but Mg^2+^ blocks transmission at resting potentials [7]. Therefore, such post-synaptic AMPAR and NMDAR-containing or purely NMDAR-containing silent synapses are functionally “deaf” [10,11,12,13]. Indeed, AMPAR has been detected in immature synapses of the brainstem nucleus but which spillover from neighboring terminals did not reach the glutamate concentration in the synaptic cleft to activate AMPAR. Additional studies have identified presynaptically non-functional synapses as a consequence of reduced rates of glutamate release (i.e., “mute” synapses), spillover or immature vesicular fusion modes (i.e, “whispering” synapses) are insufficient to activate low-affinity AMPARs [14,15,16,17]. The coexistence of mute, whispering and deaf synapses has been found at different developmental stages of most of the structures of mammalian CNS, suggesting that all of these varieties of ineffective synapses probably represent different stages of glutamatergic synapse formation (Figure 1, upper section).

Because post-synaptic NMDAR-containing silent synapses are the predominant form of a non-functional synapse, we focus here on this type of immature synapse [18,19,20,21,22]. AMPAR silent synapses have been widely found in immature neuronal networks throughout the main structures of the mammalian CNS (i.e., spinal cord [23], thalamic nuclei [24], visual cortex [25], somatosensory cortex [26,27], cerebellum [28] and hippocampal formation [8]). In all of these structures, the presence of AMPAR-containing silent synapses coincides with synaptogenesis during periods that are specific to each brain region; the number of these synapses gradually decreases as they become functional. Both immunochemical and electrophysiological studies have confirmed that most Schaffer collateral-CA1 synapses are AMPAR-silent from the first postnatal day onward; however, these synapses become practically absent from the third week of development [6,22,29,30]. Similarly, two-photon laser glutamate uncaging at single AMPAR-lacking dendritic spines induced NMDAR- but not AMPAR-mediated EPSCs; this type of response was commonly found until postnatal day 12 (P12) in somatosensory cortical pyramidal neurons. Furthermore, because focal puffing of hypertonic solution on presynaptic terminals—a common manipulation to induce neurotransmitter release—evoked NMDAR-mediated Ca^2+^ transients in the analyzed spine, the authors concluded that these AMPAR-lacking spines are contacted by release-competent presynaptic terminals [18].

In the hippocampus, from the prenatal period onward, functional heterotetrameric (GluN1/GluN2) NMDARs must contain GluN2B subunits [31,32,33,34]. Moreover, a lack of GluN2B subunits affects synaptogenesis and neonatal survival [35,36]. Moreover, ifenprodil-sensitive GluN2B-containing receptors are required for silent synapse formation; thus, this subunit constitutes a hallmark of immature synapses [37]. Near birth, NMDAR subunit composition changes from GluN2B-containing to GluN2A-containing receptors. The number of these receptors progressively increases from the first postnatal weeks in a process known as developmental switching [38,39,40]. GluN2 cytoplasmic tails are known to contain the PDZ binding motif required to drive NMDARs to spines [41]. While synaptic insertion of GluN2A-containing receptors requires APV-sensitive ligand binding more than MK-801-sensitive current flux activation, GluN2B-containing receptor delivery appears to occur independently of both channel conductance and binding site activation [31,42]. This constitutive and activity-independent insertion of GluN2B-containing NMDA receptors positions the GluN2B subunit as a putative target for the regulation of cellular processes, including soluble factor-mediated control of trafficking. Moreover, neither MCPG (which blocks metabotropic glutamate receptors) nor 5,7-dichlorokynurenic acid (a competitive antagonist at the glycine binding site) blocked synaptic delivery of GluN2B. Thus, the delivery of GluN2B-containing receptors into the synaptic membrane occurs via an activity-independent mechanism, whereas the incorporation of both GluN2A-containing receptors and AMPARs requires NMDAR activation, such as paired-depolarization long-term potentiation (LTP) [14,42]. Several studies have reported changes in the subunit composition, quantity, and regulation of AMPA receptors during activity-dependent synaptic plasticity as well as during developmental plasticity [43,44]. Homomeric GluA4-containing AMPARs are primarily expressed during the first postnatal week and are later replaced by hetero-oligomeric GluA1/2- and GluA2/3-containing receptors. GluA2-lacking, Ca^2+^-permeable AMPARs are transiently expressed during early development, but their expression is restricted to specific synapses. These receptors have been proposed as a probable route of Ca^2+^ influx during synapse maturation [45,46]. However, although the molecular mechanisms that govern the trafficking and synaptic insertion of AMPAR subunits and GluN2A-containing receptors have been well studied [40,42,47], the signals that trigger the incorporation and clustering of GluN2B-containing receptors into nascent synapses require further examination. This is especially relevant when considering the c-tail domain of GluN2B subunits. The 2B subunit contains many phosphorylation and palmitoylation regulatory sites [48], allowing for tight regulation through several mechanisms. Through this regulation, the neuron is able to incorporate NMDARs into immature synaptic contacts.

Silent synapses are also presynaptically immature, exhibiting several differences from functional synapses, which in turn differ from one synaptic circuit to another. In Sc-CA1, deaf synapses exhibit meEPSCs (miniature evoked EPSCs; recorded at +60 mV of holding potential) that contain an NMDAR-mediated component, a higher failure rate, a lack of paired-pulse facilitation (PPF), events associated with residual presynaptic calcium (see glossary) and lower synaptic potency with respect to functional synapses [19]. These silent synapses are insensitive to the conventional pharmacological tools and manipulations used to modify the probability of release (p) in functional synapses, suggesting that mechanisms that modulate intraterminal Ca^2+^ mobilization from main presynaptic Ca^2+^ stores are inactive [15,19,49,50]. In mute synapses in the hippocampus between mossy fibers and CA3 pyramidal neurons, sporadic meEPSCs that contained an AMPAR-mediated component were evoked by paired-pulse protocols (i.e., recording at a holding potential of −60 mV; AMPAR-EPSC, requiring a second pulse to obtain a response). NMDAR-mediated components with a low rate of success were also found, indicating the presence of NMDARs but alterations in the release machinery. Both components exhibited robust PPF [15] that was produced by the accumulation of residual calcium; the second pulse thus generated a larger response than the first pulse as a consequence of the low p of these synapses.

Numerous studies have described the molecular machinery available in synaptic terminals that are still being formed [51,52,53]; these studies may help to explain, at least in part, the functional properties described in electrophysiological studies. In addition, presynaptic factors, such as the endowment of voltage-gated Ca^2+^ channels, Ca^2+^ sources, Ca^2+^ buffering, synaptic vesicle pool size, and vesicle recycling have been widely studied for functional synapses [54,55,56,57,58], but little is known about the mechanisms that regulate neurotransmitter release in silent synapses.

Given the complexity of the rapid and accurate integration of different synaptic components, the transformation from an ineffective to a functional synapse must involve very tight regulation. Next, we will review electrophysiological findings concerning the conversion of glutamatergic synapses, concentrating on functional experiments carried out in slices.

## 2. How Is a Silent Synapse Converted?

- Electrophysiological properties of the conversion from a silent to a functional synapse

Depending on whether the lack of functionality is due to a pre- or post-synaptic mechanism (see above), the conversion of silent synapses into functional synapses may be induced by a wide variety of conditions and through different strategies (Figure 1). Once a new synaptic contact has been established and the initial assembly processes are complete (i.e., neurexin/neuroligin complexes) [59], NMDARs are rapidly recruited (i.e., within minutes) to sites where multiple presynaptic proteins, including synaptic vesicle precursors and active zone proteins, have previously accumulated [60]. Studies of young cortical neurons have shown that the time required for a nascent synapse to begin to release synaptic vesicles varies depending on the type of synaptic formation involved. Axodendritic contacts are presynaptically converted into functional contacts over a short time period that is coincident with NMDAR recruitment (within 15–30 min). In contrast, contacts formed by axonal growth cones are incapable of vesicle cycling for up to 1 h after the initial contact. In pairs of synaptically connected hippocampal neurons, direct stimulation of the presynaptic neuron initially fails to evoke any post-synaptic current. However, following presynaptic unsilencing, the terminals release glutamate after a brief discharge of presynaptic action potentials, generating EPSCs [1,61]. There is a growing consensus that the initiation signals that “startup” a nascent synapse should act at both the pre- and post-synaptic level (Figure 1; [62]).

In hippocampal slices from neonatal rats, Malinow and colleagues were the first to show that AMPA-containing silent synapses may be unsilenced by pairing the application of low-frequency presynaptic stimulation of Schaffer collaterals with the depolarization of post-synaptic CA1 neurons. This protocol is commonly used to induce LTP (pairing-induced LTP) [8]. As with most protocols used to induce LTP-activity dependence in functional synapses, the rapid recruitment of AMPAR to silent synapses requires post-synaptic Ca^2+^ influx through NMDARs and subsequent calcium/calmodulin-dependent protein kinase II (CaMKII) activation [21,63,64]. These studies revealed that the enhancement of synaptic efficacy in silent and functional synapses is mainly due to post-synaptic mechanisms through the insertion of functional AMPARs and does not involve presynaptic changes [64,65]. Overexpression of the protein PSD-95 (post-synaptic density-95) converts silent synapses to functional synapses, as does LTP [66]. On the other hand, PSD-95 Knock out shows a greater occurrence of silent synapses that might be related to the greater magnitude of potentiation after LTP induction observed in these mice [67]. Several components of the post-synaptic domain are key in the process of silent synapse conversion, including SynGAP, which regulates the number of silent synapses and AMPA trafficking in hippocampal and cortical neurons [68]. Direct regulation of the Glu channel has opposite consequences in the abundance of silent synapses. AMPA receptor blockade increased the number, size, and fluorescent intensity of AMPA receptor clusters and rapidly induced the appearance of AMPA receptors at silent synapses. In contrast, NMDA receptor blockade increased the size, intensity, and number of NMDA receptor clusters and decreased the number of AMPA receptor clusters, resulting in an increase in the proportion of silent synapses [12,21]. Other regulators of silent synapses presence are microRNA, post-synaptic downregulation of miR-137 at the CA3-CA1 hippocampal synapse selectively enhances AMPAR-mediated synaptic transmission and converts silent synapses to active synapses [69].

**Figure 2:**
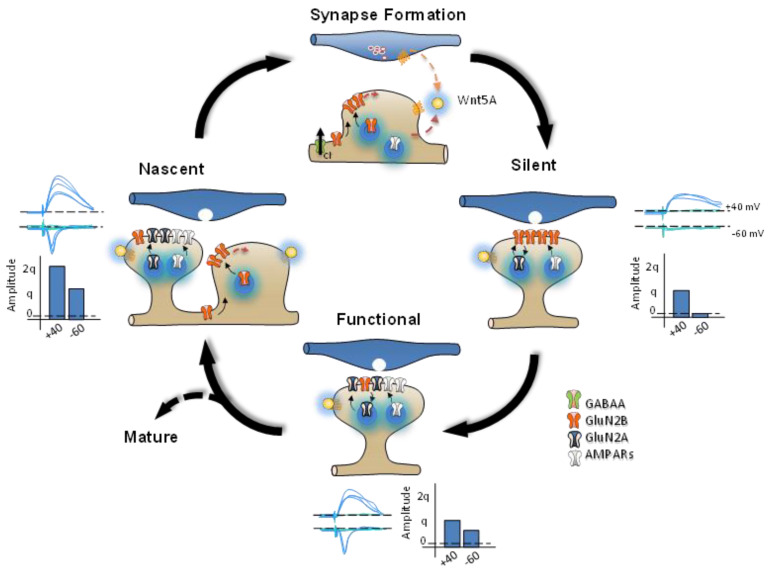
The endowment of glutamatergic receptors and functional changes during developmental plasticity. Synapse formation: GABA_A_ receptor-mediated depolarization may contribute to the release of Wnts from neurons; these Wnts can then act both pre- and postsynaptically to induce synaptogenesis and synapse conversion. Silent: At the single-fiber level, AMPA-containing silent synapses show both successes (dark blue) and failures (light blue) of synaptic transmission. At holding potentials of +40 mV and −60 mV, only NMDAR-mediated post-synaptic currents can be observed; the amplitude of these currents corresponds to q. Functional: Once they become functional, individual synapses show both failed and successful synaptic transmission. Nascent: Over a long time scale, Wnt agonists induce the birth of a new synapse. The activation of GluN2B-containing receptors at this nascent synapse can be detected as an increase in the amplitude of NMDAR, but not AMPAR currents.

Furthermore, simultaneous whole-cell recordings showed that the pairing of action potentials evoked in presynaptic neurons and the depolarization of post-synaptic neurons induced the emergence of AMPAR-EPSCs without changing the amplitude or failure rate of NMDAR-EPSCs, eliminating the possible presynaptic changes (i.e., quantal content) in this form of LTP activity-dependent (Montgomery et al., 2001). These results also confirmed that repetitive glutamate uncaging paired with post-synaptic depolarization-induced AMPAR delivery at individual thin, silent-like spines in parallel with an increase in the amplitude of AMPAR-mediated EPSCs that involved little or no NMDAR delivery [70,71]. The combination of electrophysiological studies with immunostaining for various GFP-labeled AMPA subunits in neonatal organotypic slices has shown that the endogenous neuronal activity that occurs during circuit formation is sufficient to promote the synaptic delivery of homomeric GluA4-GFP into silent contacts. Unlike the unsilencing evoked by stimulation (i.e., pairing-depolarization protocol (PDP) or high-frequency stimulation (HFS)) to induce plasticity (Figure 1), this endogenous conversion required the NMDARs but not CaMKII activation. These studies have proposed an AMPAR trafficking model based on interactions between specific AMPAR subunit compositions and the group I and II PDZ domains [43]. Several studies support the notion that GABA_A_ receptor-mediated depolarization serves as the physiological mechanism that generates sufficient depolarization to allow the activation of NMDARs that is required for the conversion of AMPAR-containing silent synapses [72,73]. Immature neurons exhibit early expression of the NKCCl chloride cotransporter, which reverses the driving force for GABA_A_ currents; as a result, GABAergic synaptic activity depolarizes post-synaptic neurons [74,75]. Recent experiments carried out both ex vivo (i.e., slices) and in vivo have confirmed that GABA_A_ -mediated depolarization during endogenous activity in developing hippocampal circuits is responsible for the activation of NMDARs in silent synapses that is required to “switch on” these silent synapses (Figure 2; [76]). Several studies suggest that the brain-derived neurotrophic factor (BDNF) serves as one of the signals involved in the presynaptic unsilencing and post-synaptic conversion of synaptic contacts. Both antibodies against BDNF and TrkB receptor inhibitors reduce presynaptic unsilencing, and presynaptic unsilencing is also reduced in the presence of APV and the Ca^2+^ chelator BAPTA. As with the model of the conversion of NMDAR-mediated post-synaptic Ca^2+^ signals, these findings strongly indicate that endogenous activity-dependent release of BDNF may also be involved in the presynaptic switching of mute synapses into those that are functional [19,77]. Below, we review the main signaling pathways that have been associated with the conversion of silent synapses, highlighting the pivotal role of Wnt signaling in inducing both the birth and conversion of silent synapses (Figure 2).

- What Triggers the Birth of Silent Synapses?

Most studies of silent synapses agree on the following key issues: the full conversion of silent synapse into a functional synapse requires the presence of NMDARs in the immature spine; their activation or recruitment is also necessary for stabilization and maturation of nascent synapse [78]. This issue raises a new question that remains unsolved: What signals trigger the birth of these NMDAR-containing-only synapses? Considerable evidence supports the involvement of several signals derived from both neurons and glia in the formation and maturation of CNS synapses, including nerve growth factor (NGF), BDNF, tumor necrosis factor-α (TNF-α), and, as recently shown, astrocyte-secreted thrombospondins (TSP) and glypicans (Gpc) (Figure 1; [79,80]).

Indeed, BDNF treatment increases the number of excitatory synapses [81]. TrkB knockout mice show lower quantities of excitatory synapses [82], whereas the amount of AMPA-containing silent synapses is increased in BDNF knockout mice [27]. However, whether BDNF-dependent mechanisms induce changes in NMDAR-mediated currents that result in the conversion of functional synapses remains controversial. BDNF induces phosphorylation of the GluN2B subunit, which increases the probability of NMDA channel opening, and this effect has been related to the increase in EPSC amplitude that BDNF induces [83,84]. In addition, BDNF increases the number of NMDARs and GABA_A_Rs located in the post-synaptic membranes of hippocampal neurons in vitro [85]. Interestingly, the increase in NMDAR clustering that is mediated by TrkB signaling is preceded by GABA_A_R clustering and requires GABA_A_R activation.

The exogenous addition of this neurotrophin to slices obtained from BDNF(−/−) mice gradually induced the emergence of AMPAR-mediated EPSCs in silent synapses, while the failure rate and synaptic potency of the NMDAR-mediated EPSCs remained unchanged [27], suggesting that BDNF alone may not be sufficient to trigger NMDAR insertion. However, several studies have reported that the conversion of AMPA-containing silent synapses that is induced by late BDNF application (i.e., >30 min) is accompanied by a rapid increase in both synaptic potency and the success rates of NMDAR-mediated EPSCs, suggesting that the delivery of both AMPARs and NMDARs may be induced by TrkB activation. Moreover, these studies showed that the unsilencing induced by both pairing-depolarization protocols and BDNF-mediated protocols can be inhibited by K252, demonstrating that endogenous BDNF may serve as a critical physiological signal that triggers developmental synaptic plasticity in the hippocampus [19,86,87,88]. Interestingly, several authors have concluded that this form of BDNF-dependent developmental plasticity may account for coordinated changes in both presynaptic and post-synaptic components, as demonstrated by an increase of p or n, respectively, which is consistent with the localization of TrkB receptors at both sides of the synapse [83,89]. Several studies have provided evidence in support of the coordinated effects of BDNF during presynaptic maturation. NMDAR activation in immature synapses can induce the release of BDNF as a retrograde messenger [77,90], which has been implicated in enhancing the accumulation and cycling of synaptic vesicles [91,92,93] as well as in developmental changes in the release machinery [19]. Similarly, two-photon laser-induced photolysis of glutamate caged at NMDAR-expressing dendritic branches of neonatal cortical neurons induces the de novo formation of functional spines, and this effect is independent of CaMKII and TrkB activation [94]. Interestingly, this study showed that new spines can also be generated by HFS and that both AMPAR- and NMDAR-mediated currents and spine Ca^2+^-transients are detectable 30 min after the application of the train of stimulation, suggesting that the nascent synapse quickly becomes functionally active. Although these findings suggest that NMDAR activation is required for the birth of new synapses, the role of BDNF as an inducer of NMDAR insertion remains controversial.

Several glia-derived factors have been described as synaptogenic signals that may contribute to the conversion of silent synapses, including apolipoprotein-E, TNF-α, TSP, hevin, and Gpc. Indeed, cytokine TNF-α released from astrocytes, the proteoglycan Gpc4-6 and, TSP1/2, an extracellular matrix protein secreted from immature astrocytes, have been associated with AMPAR recruitment, an increase of vesicle recycling, and the formation of new synapses [79,80,95,96,97]. Similar to BDNF, the main contribution of these glia-derived signals to synapse maturation involves AMPAR recruitment more than NMDAR trafficking [79] (Figure 1). Interestingly, BDNF is a Wnt signaling target gene [98], which could suggest that both signaling pathways cooperatively regulate dendritic spine formation [99]. Another example of BDNF-Wnt regulation places the Wnt/β-catenin signaling pathway downstream of BDNF stimulation controlling proliferation and differentiation of neuronal stem cells [100]. Wnt can regulate BDNF while simultaneously controlling several proteins that participate in the maturation of glutamatergic synaptic transmission. The wide variety of Wnt effects [101,102,103,104,105,106,107] position this pathway as an interesting master regulator of complex processes at the transcriptional level as well as through the rapid regulation of phosphorylation and other mechanisms.

## 3. Does Wnt signaling Trigger Silent Synapse Formation?

### 3.1. The Relevance of Wnt Signaling in the CNS

Wnt activity begins by binding Frizzled (Fz) membrane receptors, which trigger various downstream pathways, including the canonical Wnt/β-catenin control of gene transcription and the regulation of cytoskeletal proteins. A non-canonical pathway that activates Rho GTPases and c-Jun N-terminal kinase (JNK) also exists [108]. An additional non-canonical Wnt/Ca^2+^ pathway increases intracellular Ca^2+^ levels, activating calcium-sensitive kinases, such as CaMKII and PKC, two of the key enzymes that modulate synaptic efficacy. Meanwhile, the Wnt receptor Fz is a 7-transmembrane receptor that contains cysteine-rich domains (CRDs) that form the Wnt binding site [109]; 10 of these sites have been described in both mice and humans. The single-pass transmembrane receptor tyrosine kinases (RTKs) Ror1, Ror2, and Ryk [108] further increase the complexity of the activation of Wnt signaling. Several Wnt target genes are activated during this process, including c-Myc, cyclin D1, Axin2, and Ca^2+^/calmodulin-dependent protein kinase type IV (CaMKIV) [107,110,111,112]. In the absence of Wnt stimulation, cytoplasmic levels of β-catenin remain low, as this substance is ubiquitinated and constantly degraded in proteasomes [113]. “Non-canonical pathways” include ligands, such as Wnt5a and Wnt11. There are at least two of these: the planar cell polarity (PCP) pathway and the Ca^2+^ pathway. The Wnt/PCP pathway was originally identified in Drosophila, where it regulates tissue polarity and cell migration during development [114]. The PCP pathway signals through the JNK pathway to control cell polarity, which is why it is also known as the Wnt/JNK pathway [115]. During activation, Wnt binds the Fz receptors on the membrane surface, and Dishevelled (Dvl) is followed by the activation of the Rho/Rac small GTPase and JNK. Similar to Fz, Ror1-2 binds Wnts via the CRD [116,117]. The downstream effect of the activation of this pathway is the regulation of cytoskeletal organization and gene expression. Therefore, the classification of individual Wnt proteins in canonical and non-canonical terms is used here to describe the activation of signaling pathways that are either β-catenin/TCF-dependent or -independent [118,119]. Alternative Wnt pathways have been described in Drosophila Wnt-activated growth of the post-synaptic membrane is mediated by the synapse-to-nucleus translocation and active nuclear import of Fz2-C via a selective Importin-beta11/alpha2 pathway [120]. Nuclear signaling of a fragment of the Wnt-1 receptor, DFrizzled2, enters post-synaptic nuclei where it forms prominent foci, which constitute large ribonucleoprotein particles (RNPs) granules harboring synaptic protein transcripts [121].

**Figure 3:**
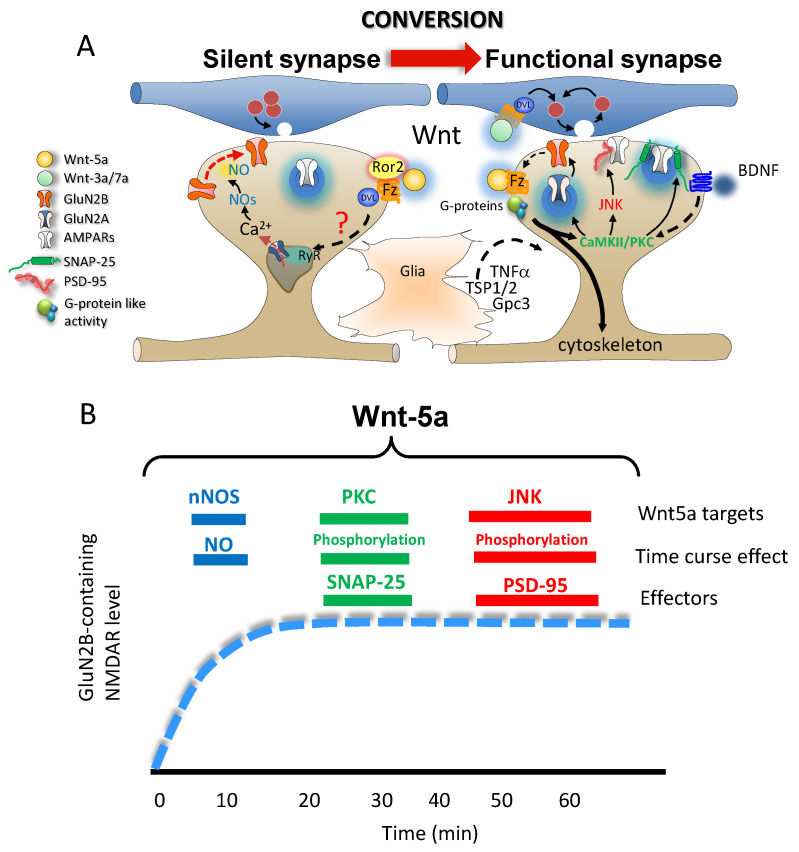
Time course of Wnt signaling activation over GluN2B-containing NMDARs. (**A**) At the post-synaptic level, the formation of silent synapses requires binding of the Wnt5a ligand to Frizzled (Fz) or Ror2 receptors; this binding activates downstream targets, such as nNOS, PKC, CaMKII, and JNK, which can modulate GluN2B-containing NMDAR translocation in synapses. The transformation of silent into functional synapse by Wnt5a ligand-mediated activation of the CaMKII/JNK and PKC/SAP-25 cascades promote the replacement of GluN2B/GluN2A-containing NMDARs and AMPAR insertion. At the presynaptic level, Wnt3a/7a ligands promote the maturation of the neurotransmitter release machinery. The G protein-like activity associated with the Fzd receptor has been identified as a modulator of cytoskeleton. Activation of this cellular pathway, in conjunction with external neuroactive factors, including BDNF, Gpc3, TNF-α, and TSP1/2, controls the conversion of silent synapses to functional synapses?: indicates the process under study. (**B**) Throughout the time course of the effects of the Wnt5a ligand on GluN2B-containing NMDARs, the activation of primary targets, including nNOS, PKC, and JNK, which are activated at different times, results in downstream activation of several effectors. NO production occurs over the shortest time period (within 5 min) and is followed by SNAP-25 phosphorylation (at approximately 20 min) and PSD-95 phosphorylation (after 1 h of Wnt ligand exposure).

Wnt signaling modulates both morphological and functional synaptic properties, and Wnt receptors are widely expressed throughout the CNS [122]. During embryonic and early postnatal development, various Wnt ligands are released both spontaneously and through activity-dependent processes, and their secretion levels vary in each brain region [123]. Tetanic stimulation induces NMDAR-dependent Wnt3a release from glutamatergic synapses in mouse hippocampal slices [124]. In cerebellar granule cells and mossy fibers, Wnt7a expression increases between P6 and P22 when synaptic contacts are made. Wnt1, Wnt3a, and Wnt5a are expressed in ventral midbrain dopaminergic neurons at the end of the embryonic stage in rats [125], whereas motoneurons of the lateral motor column express Wnt3 as they form synapses [126,127]. A growing body of evidence shows that each Wnt ligand plays a key role in the development and maturation of a given neuronal circuit [105,128,129,130], promoting specific processes, such as axon guidance [131], dendritic spinogenesis and synapse formation [132,133]. However, the importance of Wnt pathway activation in brain function has been revealed by the neuropathological effects that induce dysregulation of this pathway [122,134,135,136,137,138] as well as by its neuroprotective effects in several neurodegenerative diseases [103,139,140,141]. Moreover, Wnt plays an active role in synaptic plasticity, determining the strength and remodeling of synapses in an activity-dependent manner [102,124,139,142,143]. In addition to its widely recognized role in several developmental processes, the Wnt signaling pathway serves a wide range of key functions related to the functional maturation of synaptic contacts that enable coordinated activation of both pre- and post-synaptic mechanisms (Figure 3A). Hippocampal neurons express several Wnt ligands, including Wnt2, Wnt4, Wnt5a, Wnt7a, and Wnt11 [143,144,145]. In the time window in which most of the hippocampal circuitry is established (12-day-old animals), the production of Wnt ligand, which functionally affects the conversion process, should have a mainly neuronal origin. It has been found that neural progenitors of the hippocampus secrete Wnt3a [146], and astrocytes also participate in regulating cellular events associated with aging through the secretion of Wnt3 [147]. The mechanisms of action of the Wnt ligands can consider having a higher level of complexity. This complexity is because it is possible to detect some of the ligands in exosomes [148]. In particular, the ligands Wnt-3a, Wnt-5a, and Wnt-7a are present in exosomes derived from HT-22 neuronal cells, which secrete this type of extracellular vesicle. Incorporating these ligands into exosomes depends on the acylation process [148]. The role of acylation of Wnt ligands was also studied in primary cultures of rat hippocampal cells [149]. The authors determined that the inhibition of the enzyme Porcurin, responsible for the ligand acylation process, affects the morphology of the neurites, demonstrating the role of acylation in the morphological events mediated by Wnt ligands [149].

The Wnt7a ligand induces presynaptic protein clustering and synaptic vesicle recycling through a β-catenin-mediated pathway [144]. Further evidence has shown that in addition to its central role in axon guidance and dendrite morphogenesis, the activity-dependent release of several Wnt ligands structurally and functionally modifies dendritic branches [105,128,150]. Wnt2 has been identified as a CREB-responsive gene [151]. Accordingly, NMDAR activation and subsequent ryanodine receptor activation in the local branch were shown to induce activity-dependent dendritic growth that required CaMKI activation and triggered CREB-dependent Wnt2 transcription and expression [152]. Previous studies have shown that the cadherin/catenin complex serves as the key pathway through which extracellular signals induce dendritic morphogenesis [153,154]. In fact, increasing neuronal β-catenin levels improve dendritic arborization, whereas β-catenin sequestering decreases the number of dendritic branches. However, the dendritic growth promoted by β-catenin overexpression also reduced mEPSC amplitudes, a proposed mechanism by which synaptic strength is scaled down to prevent overexcitation during neural circuit development [155]. Taken together, these findings indicate that Fz receptor expression in pre- and post-synaptic membranes may be activated by a plethora of Wnt ligands, activating new synapse formation in a coordinated manner (Figure 3A,B).

#### 3.1.1. Control of the Presynaptic Region: The Role of Wnt

Wnt ligands have been related to assembling structural components in presynaptic compartments. By acting through a β-catenin pathway, the Wnt7a ligand induces the clustering of presynaptic proteins and the recycling of synaptic vesicles [144]. Wnt3a influences synaptic development by increasing synapsin I levels, a protein located in the presynaptic membrane and involved in the formation and function of synaptic vesicles and the induction of growth cone expansion. Both represent key events of synapse formation [105,156]. Wnt7a induces axonal spreading and the incremental growth of cone size and branching, leading to the accumulation of synaptic proteins [106,131]. Wnt7a also promotes the formation of synaptic vesicles because it increases the clustering of synapsin I [131], but it does not affect the postsynaptic clustering of proteins, such as postsynaptic density protein-95 (PSD-95), a key scaffolding protein that regulates synaptic distribution and clustering of both NMDA and AMPA receptors [144]. Moreover, Wnt7a-deficient mice exhibit fewer axonal endings and lower mEPSC frequency [101,144]. Wnt7a has been shown to modulate vesicle recycling and exocytosis [144]. Although the effects of Wnt7a correlate with β-catenin stabilization, this relationship does not involve the expression of Wnt target genes, and Wnt7a has been suggested to require Dvl1 to maintain normal synaptic vesicle recycling rates. Accordingly, the lack of both proteins (double null mutant) significantly reduces mEPSC frequency, which indicates a defect in the vesicular recycling machinery [101]. Moreover, several studies have shown that Wnt ligands induce developmental changes in presynaptic terminals, increasing synaptic efficacy. In adult rat hippocampal slices, perfusion of Wnt7a, but not Wnt5a, increased the amplitude of field excitatory postsynaptic potentials (fEPSPs) and decreased PPF in CA3-CA1 synapses [144].

Corroborating these findings, both Wnt7a and Wnt3a increased mEPSC frequency without changing mEPSC amplitude, which strongly suggests a purely presynaptic mechanism, presumably due to an increase in p [101]. Later studies of cultured hippocampal neurons showed that the Wnt3a effects required Ca^2+^ elevation in the presynaptic terminal [157]; such Ca^2+^ increases stimulate the clustering of presynaptic proteins and the exocytosis of synaptic vesicles, as revealed by FM dyes [101,144,158,159]. In fact, Wnt7a stimulates vesicle recycling and accelerates the exocytosis of synaptic vesicles. Most ligands that can modulate presynaptic differentiation have been shown to activate the Wnt/β-catenin signaling pathway. Wnt7a is also involved in the trafficking of receptors in presynaptic terminals, in increasing the number and size of co-clusters and in the trafficking of presynaptic α7-containing nicotinic acetylcholine receptors (α7-nAChR) and Adenomatous polyposis coli (APC) in hippocampal neurons [159]. These findings show that Wnt pathway components serve as a key signal about the availability of functional receptors in synaptic terminals. In addition to the role of Wnt in synaptic differentiation and function, what is the role of Fz receptors? Fz1 clusters are co-localized with presynaptic proteins, such as synapsin-1 [160], in functional synapses of cultured hippocampal neurons, and Fz1 clusters also co-localize with the post-synaptic PSD-95 scaffold [161]. Overexpression of Fz1 increases the number of clusters of a protein component of the active zone, Bassoon, suggesting that Wnt signaling also promotes active zone formation [161]. Treatment with Wnt3a, an Fz1 ligand, also induces presynaptic clustering, increasing the number of presynaptic recycling sites and the kinetics of vesicle release. These effects are prevented by incubation with the CRD of Fz1, which acts as a scavenger [161]. Thus, Wnt, by acting through Fz1, modulates synaptic differentiation and function [161]. A more detailed study has shown that Fz receptors are differentially expressed throughout hippocampal development (from E18 to P60 [160] and that some, but not all, exhibit synaptic distributions in culture. In fact, some, such as Fz7 and Fz9, localize to the soma and processes, while Fz9 and Fz5 also collect in the growth cone [160]. The specific temporal and localization patterns of Fz may increase the range of interactions that are possible during development and maturation but also adds specificity to the interactions with different Wnts. Furthermore, Wnts may serve as a common physiological signal to promote synaptic plasticity during both immature and adult stages.

In Drosophila, the presynaptic regulation includes canonical elements arrow/LRP (low-density lipoprotein receptor-related protein), disheveled, and the glycogen synthase kinase shaggy (GSK-3) and regulates the formation of microtubule loops within synaptic boutons as well as the number of synaptic boutons [162].

#### 3.1.2. Control of the Postsynaptic Region: The Role of Wnt

In the postsynaptic compartment, Wnt signaling modulates the assembly of the post-synaptic apparatus during both glutamatergic and GABAergic synaptic transmission [102,103,142]. We have shown that Wnt5a increases the amplitude of GABAAR-mediated IPSCs, which requires the recycling of functional GABAARs through a mechanism that is mediated by CAMKII activation [103]. Because GABAAR-mediated depolarization is responsible for the activation of NMDARs that is required for full conversion of silent synapses [76], Wnt ligands may serve as a key signal to control excitatory-inhibitory maturation in a coordinated manner during development.

Several findings show that both Wnt3a and Wnt5a can modulate plasticity at both developing and mature glutamatergic synapses. In cultured hippocampal neurons, we have shown that Wnt5a stimulates the de novo formation and growth of pre-existing spines through Wnt5a/Ca^2+^ pathway activation, which enhances the efficacy of the hippocampal glutamatergic synapses [143]. Indeed, an increase in intracellular levels of Wnt/β-catenin signaling or β-catenin overexpression increases the NMDAR/AMPAR ratio in parallel with increasing dendritic arborization; this effect has been interpreted as an increase in silent synapses [153,155]. We found that Wnt5a ligands increase fEPSP amplitudes, enhancing both the AMPAR and NMDAR components of EPSCs in CA3-CA1 synapses of hippocampal slices without modifying the PPF index [139,143,144]. Surprisingly, this potentiation was significantly higher in the NMDAR-mediated component of the EPSC than in the AMPAR-mediated component (i.e., 48% versus 35%, respectively). Several subsequent findings have allowed us to confirm that Wnt5a preferentially up-regulates NMDA receptors, facilitating the induction of excitatory LTP [142]. Indeed, in cultured neurons, Wnt5a induced an increase in mEPSC quantal amplitude, and this increase was approximately three times higher for the NMDAR-mediated component than for the AMPAR-mediated component. Wnt5a also increased intracellular calcium levels in dendritic processes [143]. This focal increase in calcium in synaptic puncta suggests that Wnt/Ca^2+^ signaling pathway activation is involved in a rapid phosphorylation mechanism of CaMKII. Wnt5a also modulates post-synaptic assembly, increasing PSD-95 clustering via the Wnt/JNK signaling pathway [104] without affecting total levels of PSD-95 protein or presynaptic protein clustering in cultured hippocampal neurons [104]. Supporting this finding, Wnt5a-induced enhancement of NMDAR-mediated mEPSCs occurs in two independents steps. The initial fast step is followed by slower potentiation; the first and second steps are PKC-dependent and JNK-dependent, respectively [142]. Therefore, these findings support the idea that rapid NMDAR insertion may require PKC-mediated SNAP-25 phosphorylation [163], whereas NMDAR assembly and clustering involves PSD-95 via activation of the Wnt/JNK signaling pathway [104]. Moreover, the increase in the amplitude of NMDAR-mediated miniature currents, the increase of intracellular Ca^2+^ in dendritic processes, and the de novo formation of spines that have been described in hippocampal neurons could be interpreted as the birth of silent synapses [143].

Taken together, these findings strongly suggest that Wnt signaling promotes the formation of NMDAR-rich synapses. Given that silent synapses express GluN2B-containing receptors, Wnt5a may serve as the signal responsible for the translocation and clustering of GluN2B-containing receptors in nascent synapses. In support of this notion, we showed that Wnt5a increases GluN2B subunit expression along the surface of hippocampal neurons and that this effect is mediated through nitric oxide (NO) production [164]. Indeed, the triggering of NO synthesis required activation of the non-canonical Wnt/Ca^2+^ pathway, which required Ca^2+^ release from ryanodine-sensitive intracellular stores. This mechanism is fast, occurring after 5 min of Wnt5a incubation and reaching a maximal effect at 15 min. NO production appears to be the preceding step that controls Wnt5a-mediated GluN2B insertion. The effects that occur after 20 min of treatment with Wnt5a are mediated by PKC, while the GluN2B insertion that occurs after 40 min is mediated by JNK [142] (Figure 3B). Along the same lines, our group demonstrated that Wnt5a increases potassium currents through a mechanism dependent on NO levels, which would be independent of NMDAR activation [165].

As noted above, activation of the Wnt/Ca_2+_ pathway in hippocampal neurons stimulates both dendritic spine morphogenesis and an increase in the amplitude of NMDAR-mediated spontaneous miniature currents [143]. The ability of Wnt5a to induce the localization of NMDARs into the plasma membrane of cultured hippocampal neurons increases NMDAR-mediated miniature currents, which increases glutamatergic activity via the mobilization of GluN2B NMDA receptor subunits.

The mechanism underlying Wnt-mediated regulation of NMDA receptor dynamics involves NO as an intermediary actor. Indeed, in hippocampal neurons, Wnt5a increases the number of GluN2B NMDA receptor subunits that are located on the cell surface, and this increase is mediated through NO production [164]. Work in our laboratory has demonstrated that GluN2B-containing NMDAR insertion involves three levels of Wnt5a-mediated regulation. The rapid effects of NO are followed by PKC modulation and JNK-mediated control of GluN2B insertion [142,164]. Part of the effect mediated by PKC has its origin in the activation that the ligand Wnt5a executes on the Ror2 receptor [166]. Specifically, Wnt5a participates in the depolarization of the postsynaptic neuron and increases calcium levels in dendrites [166]. This multi-target regulation of NMDAR insertion positions Wnt5a as a master switch throughout regulated GluN2B insertion. However, further studies will be necessary to establish whether the potentiation of NMDAR-mediated EPSCs that Wnt5a induces is associated with changes in the number of receptors that are inserted in a synaptic spine and/or with changes in conductance levels that are dependent on NMDAR subunit composition. Although preferential participation of the Wnt7a ligand in the regulation of molecular elements of the presynaptic zone has been demonstrated (see the previous section), there is evidence that shows that under conditions of chemical LTP and downstream of Fz7, CamKII is activated, which in turn modulates synGAP by allowing AMPARs to enter the synapse. At the same time, PKA is activated, which in turn increases the extrasynaptic AMPARs (which then move to the synapse by lateral displacement) [167]. Wnt-7a in addition to the presynaptic modulatory effects, these ligands stimulate dendritic spine morphogenesis in hippocampal cells (culture and in vivo) via glycogen synthase kinase-3 β (GSK-3β) inhibition, triggering (TCF/LEF)-dependent gene transcription and promoting postsynaptic density-95 (PSD-95) protein expression. This modulatory effect results after at least three hours of treatment [168]. In addition, at the transcriptional level, the Wnt-5a ligand can increase the levels of NMDA-type receptors, particularly the GluN2B subunit [169]. This effect is complemented by what was observed at shorter times and described above. GluN2B levels increase in response to Wnt-5 through a mechanism involving Heme-regulated eukaryotic initiation factor 2α (HRI) kinase and transcription initiator factor eIF2α [169]. These specific transcriptional events on postsynaptic components remain under the umbrella of broader modulatory effects. Wnt-3a treatment, such as the activation of GSK-3β by lithium, generates a series of changes in mRNA and protein levels at the pre- and post-synaptic level [170]. These changes show a particular temporality, where presynaptic changes precede changes in PSD-95, when the canonical Wnt pathway is modulated [170].

## 4. A Model for Silent Synapse Conversion

Our model proposes that Wnt pathway activation serves as the pivotal signaling mechanism that controls several events associated with synaptic development and maturation, including inducing the birth of synapses and plastic remodeling. These events require synchronization between presynaptic and postsynaptic components on both sides of the synaptic cleft. Wnt signaling modulates various processes (see above). However, more importantly, these events are controlled by multiple pathways. For example, the regulation of calcium homeostasis involves the Wnt/Ca^2+^ pathway, cytoskeleton remodeling involves Wnt/β-catenin, and changes in NO levels involve non-canonical activation. The characteristic targets of Wnt activation, including PKC, JNK, and β-catenin, are also involved in other cellular pathways that participate in important synaptic events (e.g., synaptic vesicle release, receptor insertion, and scaffolding protein reorganization).

The wide array of events that are regulated by Wnt signaling includes the insertion of receptors [102,103,142,171], the control of cytoskeletal dynamics [133], the regulation of postsynaptic scaffolding proteins [104], synaptic vesicle movement and the regulation of presynaptic vesicle release [144], indicating that the Wnt family serves as a very attractive candidate for a master switch that controls the birth and conversion of silent synapses. However, more importantly, Wnt signaling closely regulates well-characterized factors that can control, at least in part, these cellular events. BDNF is a Wnt target gene, but other soluble factors that share functions with BDNF may be regulated by Wnt during the birth of new synapses or the conversion from silent to functional synapses, which controls glutamate receptor dynamics [172].

Recent evidence suggests that phosphoinositide (PIP) signaling, such as the direction of MIM proteins (I-BAR and IF-BAR) in the generation of proto-protrusions, promotes actin assembly, which is then followed by the initiation of dendritic spines in neurons [173]. The role of actin in the development of filopodia-like structures that occurs prior to the formation of mature synaptic spines is well characterized [174]. MIM works as a nucleator of dendritic spines and accumulates at sites of future spine initiation in a PIP2-dependent manner [173]. It is interesting that growth factors, such as BDNF promote PIP signaling [175], but, more important, PIP signaling is regulated by the Wnt signaling pathway [176,177], and direct or indirect Wnt-mediated regulation of PIP signaling appears to control the formation of nascent new spines in steps that precede the regulation of actin polymerization. The multiple branches of the Wnt pathway enable regulation through crosstalk with proteins at the level of membrane receptors. The role of LRP-6 as such a regulator of PIP signaling provides a representative example of this [177], as well as its regulation of Fz receptors [178].

When we explore the temporal and spatial aspects of Wnt-mediated regulation of specific targets to control glutamate receptor dynamics more deeply, it is possible to identify specific components for their temporal and spatial fine-tuning of these effects. At a short time scale, Wnt increases NO, which leads to subsequent activation of PKC and, even later, the activation of JNK. Together, these changes significantly modify synaptic communication by controlling GluN2B-containing NMDAR insertion (Figure 3B). Additionally, Wnt activation also controls events over a longer time scale. Once organisms are born, Wnt release facilitates the initial crosstalk that occurs between pre- and postsynaptic components by directly regulating vesicle release, as well as the insertion of post-synaptic receptors. Later, similar processes are regulated by Wnt activation in mature synapses, such as vesicle release and the insertion of postsynaptic receptors, allowing the reorganization of both pre-and postsynaptic components with clear implications for synaptic plasticity (functional and structural; Figure 2). In this case, the pre-post synaptic crosstalk controlled by Wnts involves a third factor, glial cells, which may contribute to the tight regulation necessary for synaptic functional transformation. In the regulation of synaptic homeostasis, including the maintenance of glutamatergic transmission, the proper functioning of autophagy is essential [179]. Therefore, regulation of autophagy is relevant in the context of synaptic plasticity. The Wnt-3a ligand can promote the rapid activation of AMPK through the inactivation of GSK-3β [180]. This activation modulates autophagy in hippocampal neurons [180]. Most of the alternatives for activating the Wnt pathway include binding the ligand to the Fzd receptor. This receptor corresponds to an unconventional G protein-coupled receptor (GPCR) and has been identified as having activity of this type [181]. The G protein-like activity associated with the Fzd receptor has been identified as a modulator of the dendritic spine induction process controlling cytoskeleton [182,183] (Figure 3A).

An attractive hypothesis is that glia releases soluble factors in response to Wnt signaling; such factors include TNF-α, TSP1-2, hevin, and Gpc, which might induce AMPAR insertion and dendritic remodeling even when a synapse is not yet functional (Figure 3). To test this hypothesis, the following questions should be addressed: Who releases Wnt? What signals trigger the release of Wnt? Is the calcium influx through the GluN2B-containing NMDA receptor necessary for Wnt-dependent conversion from silent to functional synapses? Further studies will be necessary to further characterize the relationships between Wnt signaling and glial cells, as well as their putative roles in synaptic maturation.

## 5. Concluding Remarks

Activation of Wnt signaling produces a wide range of effects on CNS synapses [102,105,116,128,139,184]. We present recent evidence in support of the role of Wnt5a in controlling structural and functional changes in central synapses, with a specific focus on functional/electrophysiological experiments and with an initial exploration of potential contributions of quantal theory in bridging structural and functional approaches. The evidence positions Wnt signaling as a possible master key in the control of the birth and conversion of silent synapses. Consistent evidence shows that Wnt5a increases the quantity of GluN2B-containing NMDARs, which serve as a central player in the birth of glutamatergic synapses as well as in the conversion of silent synapses. In this review, we present evidence indicating that Wnt pathway activation, specifically by the Wnt5a ligand, induces the incorporation of NMDA receptors into synaptic membranes through a mechanism that involves several targets, such as nNOS, PKC, and JNK. Wnt5a increases the number of GluN2B-containing NMDARs that are found in the membrane on the scale of minutes [102,142,164]. NO production, PKC activation, and JNK modulation mediate the regulation of NMDAR levels. We recently reported that Ror2 is responsible for Wnt-regulated insertion of NMDA receptors [102], and we have also reported on the role of the Fz receptor [143]. This same Wnt5a pathway may contribute to the Ca^2+^ elevation that is required for the conversion of GluN2B-containing NMDA receptors and silent synapses; the Ca^2+^ elevation then activates CaMKII/scaffolding protein pathways for AMPAR insertion into the synaptic membrane.

This complex overall picture, which involves a variety of targets and receptors, suggests that activation of the Wnt signaling pathway may serve as a central player in the regulation of NMDA receptors and other synaptic components involved in the birth and conversion of silent synapses in the CNS.

## Data Availability

Not applicable.

## References

[1] McAllister A.K. (2007). Dynamic aspects of CNS synapse formation. Annu. Rev. Neurosci..

[2] Li Z., Sheng M. (2003). Some assembly required: The development of neuronal synapses. Nat. Rev. Mol. Cell Biol..

[3] Semyanov A., Verkhratsky A. (2021). Astrocytic processes: From tripartite synapses to the active milieu. Trends Neurosci..

[4] Groc L., Gustafsson B., Hanse E. (2003). Early establishment of multiple release site connectivity between interneurons and pyramidal neurons in the developing hippocampus. Eur. J. Neurosci..

[5] Hanse E., Gustafsson B. (2001). Quantal variability at glutamatergic synapses in area CA1 of the rat neonatal hippocampus. J. Physiol..

[6] Hsia A.Y., Malenka R.C., Nicoll R.A. (1998). Development of excitatory circuitry in the hippocampus. J. Neurophysiol..

[7] Isaac J.T., Nicoll R.A., Malenka R.C. (1995). Evidence for silent synapses: Implications for the expression of LTP. Neuron.

[8] Liao D., Hessler N.A., Malinow R. (1995). Activation of postsynaptically silent synapses during pairing-induced LTP in CA1 region of hippocampal slice. Nature.

[9] Raastad M., Storm J.F., Andersen P. (1992). Putative Single Quantum and Single Fibre Excitatory Postsynaptic Currents Show Similar Amplitude Range and Variability in Rat Hippocampal Slices. Eur. J. Neurosci..

[10] Groc L., Gustafsson B., Hanse E. (2006). AMPA signalling in nascent glutamatergic synapses: There and not there!. Trends Neurosci..

[11] Hanse E., Taira T., Lauri S., Groc L. (2009). Glutamate synapse in developing brain: An integrative perspective beyond the silent state. Trends Neurosci..

[12] Liao D., Zhang X., O'Brien R., Ehlers M.D., Huganir R.L. (1999). Regulation of morphological postsynaptic silent synapses in developing hippocampal neurons. Nat. Neurosci..

[13] Pickard L., Noel J., Duckworth J.K., Fitzjohn S.M., Henley J.M., Collingridge G.L., Molnar E. (2001). Transient synaptic activation of NMDA receptors leads to the insertion of native AMPA receptors at hippocampal neuronal plasma membranes. Neuropharmacology.

[14] Choi S., Klingauf J., Tsien R.W. (2000). Postfusional regulation of cleft glutamate concentration during LTP at ‘silent synapses’. Nat. Neurosci..

[15] Gasparini S., Saviane C., Voronin L.L., Cherubini E. (2000). Silent synapses in the developing hippocampus: Lack of functional AMPA receptors or low probability of glutamate release?. Proc. Natl. Acad. Sci. USA.

[16] Voronin L.L., Cherubini E. (2004). 'Deaf, mute and whispering' silent synapses: Their role in synaptic plasticity. J. Physiol..

[17] Balland B., Lachamp P., Kessler J.P., Tell F. (2008). Silent synapses in developing rat nucleus tractus solitarii have AMPA receptors. J. Neurosci. Off. J. Soc. Neurosci..

[18] Busetto G., Higley M.J., Sabatini B.L. (2008). Developmental presence and disappearance of postsynaptically silent synapses on dendritic spines of rat layer 2/3 pyramidal neurons. J. Physiol..

[19] Cabezas C., Buno W. (2006). Distinct transmitter release properties determine differences in short-term plasticity at functional and silent synapses. J. Neurophysiol..

[20] Kerchner G.A., Nicoll R.A. (2008). Silent synapses and the emergence of a postsynaptic mechanism for LTP. Nat. Rev. Neurosci..

[21] Liao D., Scannevin R.H., Huganir R. (2001). Activation of silent synapses by rapid activity-dependent synaptic recruitment of AMPA receptors. J. Neurosci..

[22] Petralia R.S., Esteban J.A., Wang Y.X., Partridge J.G., Zhao H.M., Wenthold R.J., Malinow R. (1999). Selective acquisition of AMPA receptors over postnatal development suggests a molecular basis for silent synapses. Nat. Neurosci..

[23] Yasaka T., Hughes D.I., Polgar E., Nagy G.G., Watanabe M., Riddell J.S., Todd A.J. (2009). Evidence against AMPA receptor-lacking glutamatergic synapses in the superficial dorsal horn of the rat spinal cord. J. Neurosci. Off. J. Soc. Neurosci..

[24] Chen C., Regehr W.G. (2000). Developmental remodeling of the retinogeniculate synapse. Neuron.

[25] Rumpel S., Hatt H., Gottmann K. (1998). Silent synapses in the developing rat visual cortex: Evidence for postsynaptic expression of synaptic plasticity. J. Neurosci. Off. J. Soc. Neurosci..

[26] Isaac J.T., Crair M.C., Nicoll R.A., Malenka R.C. (1997). Silent synapses during development of thalamocortical inputs. Neuron.

[27] Itami C., Kimura F., Kohno T., Matsuoka M., Ichikawa M., Tsumoto T., Nakamura S. (2003). Brain-derived neurotrophic factor-dependent unmasking of "silent" synapses in the developing mouse barrel cortex. Proc. Natl. Acad. Sci. USA.

[28] Losi G., Prybylowski K., Fu Z., Luo J.H., Vicini S. (2002). Silent synapses in developing cerebellar granule neurons. J. Neurophysiol..

[29] Nusser Z., Lujan R., Laube G., Roberts J.D., Molnar E., Somogyi P. (1998). Cell type and pathway dependence of synaptic AMPA receptor number and variability in the hippocampus. Neuron.

[30] Racca C., Stephenson F.A., Streit P., Roberts J.D., Somogyi P. (2000). NMDA receptor content of synapses in stratum radiatum of the hippocampal CA1 area. J. Neurosci. Off. J. Soc. Neurosci..

[31] Barria A., Malinow R. (2002). Subunit-specific NMDA receptor trafficking to synapses. Neuron.

[32] Sheng M. (2001). The postsynaptic NMDA-receptor--PSD-95 signaling complex in excitatory synapses of the brain. J. Cell Sci..

[33] Standley S., Roche K.W., McCallum J., Sans N., Wenthold R.J. (2000). PDZ domain suppression of an ER retention signal in NMDA receptor NR1 splice variants. Neuron.

[34] Xia H., Hornby Z.D., Malenka R.C. (2001). An ER retention signal explains differences in surface expression of NMDA and AMPA receptor subunits. Neuropharmacology.

[35] Kutsuwada T., Sakimura K., Manabe T., Takayama C., Katakura N., Kushiya E., Natsume R., Watanabe M., Inoue Y., Yagi T. (1996). Impairment of suckling response, trigeminal neuronal pattern formation, and hippocampal LTD in NMDA receptor epsilon 2 subunit mutant mice. Neuron.

[36] Westbrook G.L., Krupp J.J., Vissel B. (1997). Cytoskeletal interactions with glutamate receptors at central synapses. Soc. Gen. Physiol. Ser..

[37] Nakayama K., Kiyosue K., Taguchi T. (2005). Diminished neuronal activity increases neuron-neuron connectivity underlying silent synapse formation and the rapid conversion of silent to functional synapses. J. Neurosci..

[38] Monyer H., Burnashev N., Laurie D.J., Sakmann B., Seeburg P.H. (1994). Developmental and regional expression in the rat brain and functional properties of four NMDA receptors. Neuron.

[39] Sheng M. (2001). Molecular organization of the postsynaptic specialization. Proc. Natl. Acad. Sci. USA.

[40] van Zundert B., Yoshii A., Constantine-Paton M. (2004). Receptor compartmentalization and trafficking at glutamate synapses: A developmental proposal. Trends Neurosci..

[41] Garner C.C., Nash J., Huganir R.L. (2000). PDZ domains in synapse assembly and signalling. Trends Cell Biol..

[42] Storey G.P., Opitz-Araya X., Barria A. (2011). Molecular determinants controlling NMDA receptor synaptic incorporation. J. Neurosci..

[43] Shi S., Hayashi Y., Esteban J.A., Malinow R. (2001). Subunit-specific rules governing AMPA receptor trafficking to synapses in hippocampal pyramidal neurons. Cell.

[44] Zhu J.J. (2003). Mechanisms of synaptic plasticity: From membrane to intracellular AMPAR trafficking. Mol. Interv..

[45] Ho M.T., Pelkey K.A., Topolnik L., Petralia R.S., Takamiya K., Xia J., Huganir R.L., Lacaille J.C., McBain C.J. (2007). Developmental expression of Ca^2+^-permeable AMPA receptors underlies depolarization-induced long-term depression at mossy fiber CA3 pyramid synapses. J. Neurosci. Off. J. Soc. Neurosci..

[46] Liu S.Q., Cull-Candy S.G. (2000). Synaptic activity at calcium-permeable AMPA receptors induces a switch in receptor subtype. Nature.

[47] Kubota S., Kitajima T. (2008). A model for synaptic development regulated by NMDA receptor subunit expression. J. Comput. Neurosci..

[48] Traynelis S.F., Wollmuth L.P., McBain C.J., Menniti F.S., Vance K.M., Ogden K.K., Hansen K.B., Yuan H., Myers S.J., Dingledine R. (2010). Glutamate receptor ion channels: Structure, regulation, and function. Pharm. Rev..

[49] Emptage N.J., Reid C.A., Fine A. (2001). Calcium stores in hippocampal synaptic boutons mediate short-term plasticity, store-operated Ca^2+^ entry, and spontaneous transmitter release. Neuron.

[50] Fernandez de Sevilla D., Cabezas C., de Prada A.N., Sanchez-Jimenez A., Buno W. (2002). Selective muscarinic regulation of functional glutamatergic Schaffer collateral synapses in rat CA1 pyramidal neurons. J. Physiol..

[51] Ahmari S.E., Buchanan J., Smith S.J. (2000). Assembly of presynaptic active zones from cytoplasmic transport packets. Nat. Neurosci..

[52] Zhai R.G., Bellen H.J. (2004). The architecture of the active zone in the presynaptic nerve terminal. Physiology.

[53] Zhai R.G., Vardinon-Friedman H., Cases-Langhoff C., Becker B., Gundelfinger E.D., Ziv N.E., Garner C.C. (2001). Assembling the presynaptic active zone: A characterization of an active one precursor vesicle. Neuron.

[54] Dobrunz L.E. (2002). Release probability is regulated by the size of the readily releasable vesicle pool at excitatory synapses in hippocampus. Int. J. Dev. Neurosci..

[55] Hua Y., Sinha R., Thiel C.S., Schmidt R., Huve J., Martens H., Hell S.W., Egner A., Klingauf J. (2011). A readily retrievable pool of synaptic vesicles. Nat. Neurosci..

[56] Saviane C., Silver R.A. (2006). Fast vesicle reloading and a large pool sustain high bandwidth transmission at a central synapse. Nature.

[57] Scheuber A., Miles R., Poncer J.C. (2004). Presynaptic Cav2.1 and Cav2.2 differentially influence release dynamics at hippocampal excitatory synapses. J. Neurosci. Off. J. Soc. Neurosci..

[58] Scott R., Rusakov D.A. (2008). Ca^2+^ stores and use-dependent facilitation of presynaptic Ca^2+^ signaling. Proc. Natl. Acad. Sci. USA.

[59] Varoqueaux F., Aramuni G., Rawson R.L., Mohrmann R., Missler M., Gottmann K., Zhang W., Sudhof T.C., Brose N. (2006). Neuroligins determine synapse maturation and function. Neuron.

[60] Washbourne P., Bennett J.E., McAllister A.K. (2002). Rapid recruitment of NMDA receptor transport packets to nascent synapses. Nat. Neurosci..

[61] Tada T., Sheng M. (2006). Molecular mechanisms of dendritic spine morphogenesis. Curr. Opin. Neurobiol..

[62] Chavis P., Westbrook G. (2001). Integrins mediate functional pre- and postsynaptic maturation at a hippocampal synapse. Nature.

[63] Hayashi Y., Shi S.H., Esteban J.A., Piccini A., Poncer J.C., Malinow R. (2000). Driving AMPA receptors into synapses by LTP and CaMKII: Requirement for GluR1 and PDZ domain interaction. Science.

[64] Isaac J.T., Oliet S.H., Hjelmstad G.O., Nicoll R.A., Malenka R.C. (1996). Expression mechanisms of long-term potentiation in the hippocampus. J. Physiol. Paris.

[65] Goda Y., Stevens C.F. (1996). Synaptic plasticity: The basis of particular types of learning. Curr. Biol..

[66] Stein V., House D.R., Bredt D.S., Nicoll R.A. (2003). Postsynaptic density-95 mimics and occludes hippocampal long-term potentiation and enhances long-term depression. J. Neurosci. Off. J. Soc. Neurosci..

[67] Beique J.C., Lin D.T., Kang M.G., Aizawa H., Takamiya K., Huganir R.L. (2006). Synapse-specific regulation of AMPA receptor function by PSD-95. Proc. Natl. Acad. Sci. USA.

[68] Rumbaugh G., Adams J.P., Kim J.H., Huganir R.L. (2006). SynGAP regulates synaptic strength and mitogen-activated protein kinases in cultured neurons. Proc. Natl. Acad. Sci. USA.

[69] Olde Loohuis N.F., Ba W., Stoerchel P.H., Kos A., Jager A., Schratt G., Martens G.J., van Bokhoven H., Nadif Kasri N., Aschrafi A. (2015). MicroRNA-137 Controls AMPA-Receptor-Mediated Transmission and mGluR-Dependent LTD. Cell Rep..

[70] Matsuzaki M., Ellis-Davies G.C., Nemoto T., Miyashita Y., Iino M., Kasai H. (2001). Dendritic spine geometry is critical for AMPA receptor expression in hippocampal CA1 pyramidal neurons. Nat. Neurosci..

[71] Matsuzaki M., Honkura N., Ellis-Davies G.C., Kasai H. (2004). Structural basis of long-term potentiation in single dendritic spines. Nature.

[72] Khazipov R., Leinekugel X., Khalilov I., Gaiarsa J.L., Ben-Ari Y. (1997). Synchronization of GABAergic interneuronal network in CA3 subfield of neonatal rat hippocampal slices. J. Physiol..

[73] Wang D.D., Kriegstein A.R. (2008). GABA regulates excitatory synapse formation in the neocortex via NMDA receptor activation. J. Neurosci. Off. J. Soc. Neurosci..

[74] Sipila S.T., Schuchmann S., Voipio J., Yamada J., Kaila K. (2006). The cation-chloride cotransporter NKCC1 promotes sharp waves in the neonatal rat hippocampus. J. Physiol..

[75] Pfeffer C.K., Stein V., Keating D.J., Maier H., Rinke I., Rudhard Y., Hentschke M., Rune G.M., Jentsch T.J., Hubner C.A. (2009). NKCC1-dependent GABAergic excitation drives synaptic network maturation during early hippocampal development. J. Neurosci. Off. J. Soc. Neurosci..

[76] Chancey J.H., Adlaf E.W., Sapp M.C., Pugh P.C., Wadiche J.I., Overstreet-Wadiche L.S. (2013). GABA depolarization is required for experience-dependent synapse unsilencing in adult-born neurons. J. Neurosci. Off. J. Soc. Neurosci..

[77] Shen W., Wu B., Zhang Z., Dou Y., Rao Z.R., Chen Y.R., Duan S. (2006). Activity-induced rapid synaptic maturation mediated by presynaptic cdc42 signaling. Neuron.

[78] Zito K., Scheuss V., Knott G., Hill T., Svoboda K. (2009). Rapid functional maturation of nascent dendritic spines. Neuron.

[79] Allen N.J., Bennett M.L., Foo L.C., Wang G.X., Chakraborty C., Smith S.J., Barres B.A. (2012). Astrocyte glypicans 4 and 6 promote formation of excitatory synapses via GluA1 AMPA receptors. Nature.

[80] Christopherson K.S., Ullian E.M., Stokes C.C., Mullowney C.E., Hell J.W., Agah A., Lawler J., Mosher D.F., Bornstein P., Barres B.A. (2005). Thrombospondins are astrocyte-secreted proteins that promote CNS synaptogenesis. Cell.

[81] Hale C.F., Dietz K.C., Varela J.A., Wood C.B., Zirlin B.C., Leverich L.S., Greene R.W., Cowan C.W. (2011). Essential role for vav Guanine nucleotide exchange factors in brain-derived neurotrophic factor-induced dendritic spine growth and synapse plasticity. J. Neurosci. Off. J. Soc. Neurosci..

[82] Martinez A., Alcantara S., Borrell V., Del Rio J.A., Blasi J., Otal R., Campos N., Boronat A., Barbacid M., Silos-Santiago I. (1998). TrkB and TrkC signaling are required for maturation and synaptogenesis of hippocampal connections. J. Neurosci. Off. J. Soc. Neurosci..

[83] Alder J., Thakker-Varia S., Crozier R.A., Shaheen A., Plummer M.R., Black I.B. (2005). Early presynaptic and late postsynaptic components contribute independently to brain-derived neurotrophic factor-induced synaptic plasticity. J. Neurosci. Off. J. Soc. Neurosci..

[84] Lin S.Y., Wu K., Levine E.S., Mount H.T., Suen P.C., Black I.B. (1998). BDNF acutely increases tyrosine phosphorylation of the NMDA receptor subunit 2B in cortical and hippocampal postsynaptic densities. Brain Res. Mol. Brain Res..

[85] Elmariah S.B., Crumling M.A., Parsons T.D., Balice-Gordon R.J. (2004). Postsynaptic TrkB-mediated signaling modulates excitatory and inhibitory neurotransmitter receptor clustering at hippocampal synapses. J. Neurosci. Off. J. Soc. Neurosci..

[86] Kolarow R., Brigadski T., Lessmann V. (2007). Postsynaptic secretion of BDNF and NT-3 from hippocampal neurons depends on calcium calmodulin kinase II signaling and proceeds via delayed fusion pore opening. J. Neurosci. Off. J. Soc. Neurosci..

[87] Yoshii A., Constantine-Paton M. (2007). BDNF induces transport of PSD-95 to dendrites through PI3K-AKT signaling after NMDA receptor activation. Nat. Neurosci..

[88] Yoshii A., Constantine-Paton M. (2014). Postsynaptic localization of PSD-95 is regulated by all three pathways downstream of TrkB signaling. Front. Synaptic. Neurosci..

[89] Madara J.C., Levine E.S. (2008). Presynaptic and postsynaptic NMDA receptors mediate distinct effects of brain-derived neurotrophic factor on synaptic transmission. J. Neurophysiol..

[90] Tao H.W., Poo M. (2001). Retrograde signaling at central synapses. Proc. Natl. Acad. Sci. USA.

[91] Hanse E., Gustafsson B. (2001). Vesicle release probability and pre-primed pool at glutamatergic synapses in area CA1 of the rat neonatal hippocampus. J. Physiol..

[92] Tyler W.J., Zhang X.L., Hartman K., Winterer J., Muller W., Stanton P.K., Pozzo-Miller L. (2006). BDNF increases release probability and the size of a rapidly recycling vesicle pool within rat hippocampal excitatory synapses. J. Physiol..

[93] Walz C., Jungling K., Lessmann V., Gottmann K. (2006). Presynaptic plasticity in an immature neocortical network requires NMDA receptor activation and BDNF release. J. Neurophysiol..

[94] Kwon H.B., Sabatini B.L. (2011). Glutamate induces de novo growth of functional spines in developing cortex. Nature.

[95] Beattie E.C., Stellwagen D., Morishita W., Bresnahan J.C., Ha B.K., Von Zastrow M., Beattie M.S., Malenka R.C. (2002). Control of synaptic strength by glial TNFalpha. Science.

[96] Mauch D.H., Nagler K., Schumacher S., Goritz C., Muller E.C., Otto A., Pfrieger F.W. (2001). CNS synaptogenesis promoted by glia-derived cholesterol. Science.

[97] Hennekinne L., Colasse S., Triller A., Renner M. (2013). Differential control of thrombospondin over synaptic glycine and AMPA receptors in spinal cord neurons. J. Neurosci. Off. J. Soc. Neurosci..

[98] Yi H., Hu J., Qian J., Hackam A.S. (2012). Expression of brain-derived neurotrophic factor is regulated by the Wnt signaling pathway. Neuroreport.

[99] Hiester B.G., Galati D.F., Salinas P.C., Jones K.R. (2013). Neurotrophin and Wnt signaling cooperatively regulate dendritic spine formation. Mol. Cell. Neurosci..

[100] Chen B.Y., Wang X., Wang Z.Y., Wang Y.Z., Chen L.W., Luo Z.J. (2013). Brain-derived neurotrophic factor stimulates proliferation and differentiation of neural stem cells, possibly by triggering the Wnt/beta-catenin signaling pathway. J. Neurosci. Res..

[101] Ahmad-Annuar A., Ciani L., Simeonidis I., Herreros J., Fredj N.B., Rosso S.B., Hall A., Brickley S., Salinas P.C. (2006). Signaling across the synapse: A role for Wnt and Dishevelled in presynaptic assembly and neurotransmitter release. J. Cell Biol..

[102] Cerpa W., Latorre-Esteves E., Barria A. (2015). RoR2 functions as a noncanonical Wnt receptor that regulates NMDAR-mediated synaptic transmission. Proc. Natl. Acad. Sci. USA.

[103] Cuitino L., Godoy J.A., Farias G.G., Couve A., Bonansco C., Fuenzalida M., Inestrosa N.C. (2010). Wnt-5a modulates recycling of functional GABAA receptors on hippocampal neurons. J. Neurosci. Off. J. Soc. Neurosci..

[104] Farias G.G., Alfaro I.E., Cerpa W., Grabowski C.P., Godoy J.A., Bonansco C., Inestrosa N.C. (2009). Wnt-5a/JNK signaling promotes the clustering of PSD-95 in hippocampal neurons. J. Biol. Chem..

[105] Inestrosa N.C., Arenas E. (2010). Emerging roles of Wnts in the adult nervous system. Nat. Rev. Neurosci..

[106] Lucas F.R., Salinas P.C. (1997). WNT-7a induces axonal remodeling and increases synapsin I levels in cerebellar neurons. Dev. Biol..

[107] Nusse R., Varmus H. (2012). Three decades of Wnts: A personal perspective on how a scientific field developed. EMBO J..

[108] Gordon M.D., Nusse R. (2006). Wnt signaling: Multiple pathways, multiple receptors, and multiple transcription factors. J. Biol. Chem..

[109] Bhanot P., Brink M., Samos C.H., Hsieh J.C., Wang Y., Macke J.P., Andrew D., Nathans J., Nusse R. (1996). A new member of the frizzled family from Drosophila functions as a Wingless receptor. Nature.

[110] Arrazola M.S., Varela-Nallar L., Colombres M., Toledo E.M., Cruzat F., Pavez L., Assar R., Aravena A., Gonzalez M., Montecino M. (2009). Calcium/calmodulin-dependent protein kinase type IV is a target gene of the Wnt/beta-catenin signaling pathway. J. Cell. Physiol..

[111] Hodar C., Assar R., Colombres M., Aravena A., Pavez L., Gonzalez M., Martinez S., Inestrosa N.C., Maass A. (2010). Genome-wide identification of new Wnt/beta-catenin target genes in the human genome using CART method. BMC Genom..

[112] Toledo E.M., Colombres M., Inestrosa N.C. (2008). Wnt signaling in neuroprotection and stem cell differentiation. Prog. Neurobiol..

[113] Aberle H., Bauer A., Stappert J., Kispert A., Kemler R. (1997). beta-catenin is a target for the ubiquitin-proteasome pathway. EMBO J..

[114] Veeman M.T., Axelrod J.D., Moon R.T. (2003). A second canon. Functions and mechanisms of beta-catenin-independent Wnt signaling. Dev. Cell..

[115] Boutros M., Paricio N., Strutt D.I., Mlodzik M. (1998). Dishevelled activates JNK and discriminates between JNK pathways in planar polarity and wingless signaling. Cell.

[116] Logan C.Y., Nusse R. (2004). The Wnt signaling pathway in development and disease. Annu. Rev. Cell. Dev. Biol..

[117] Grumolato L., Liu G., Mong P., Mudbhary R., Biswas R., Arroyave R., Vijayakumar S., Economides A.N., Aaronson S.A. (2010). Canonical and noncanonical Wnts use a common mechanism to activate completely unrelated coreceptors. Genes Dev..

[118] van Amerongen R., Nusse R. (2009). Towards an integrated view of Wnt signaling in development. Development.

[119] Mikels A.J., Nusse R. (2006). Purified Wnt5a protein activates or inhibits beta-catenin-TCF signaling depending on receptor context. PLoS Biol..

[120] Mosca T.J., Schwarz T.L. (2010). The nuclear import of Frizzled2-C by Importins-beta11 and alpha2 promotes postsynaptic development. Nat. Neurosci..

[121] Speese S.D., Ashley J., Jokhi V., Nunnari J., Barria R., Li Y., Ataman B., Koon A., Chang Y.T., Li Q. (2012). Nuclear envelope budding enables large ribonucleoprotein particle export during synaptic Wnt signaling. Cell.

[122] Inestrosa N.C., Varela-Nallar L. (2014). Wnt signaling in the nervous system and in Alzheimer's disease. J. Mol. Cell Biol..

[123] Oliva C.A., Vargas J.Y., Inestrosa N.C. (2013). Wnts in adult brain: From synaptic plasticity to cognitive deficiencies. Front. Cell. Neurosci..

[124] Chen J., Park C.S., Tang S.J. (2006). Activity-dependent synaptic Wnt release regulates hippocampal long term potentiation. J. Biol. Chem..

[125] Castelo-Branco G., Wagner J., Rodriguez F.J., Kele J., Sousa K., Rawal N., Pasolli H.A., Fuchs E., Kitajewski J., Arenas E. (2003). Differential regulation of midbrain dopaminergic neuron development by Wnt-1, Wnt-3a, and Wnt-5a. Proc. Natl. Acad. Sci. USA.

[126] Krylova O., Herreros J., Cleverley K.E., Ehler E., Henriquez J.P., Hughes S.M., Salinas P.C. (2002). WNT-3, expressed by motoneurons, regulates terminal arborization of neurotrophin-3-responsive spinal sensory neurons. Neuron.

[127] Pinto C., Perez V., Mella J., Albistur M., Caprile T., Bronfman F.C., Henriquez J.P. (2021). Transport and Secretion of the Wnt3 Ligand by Motor Neuron-like Cells and Developing Motor Neurons. Biomolecules.

[128] Ciani L., Salinas P.C. (2005). WNTs in the vertebrate nervous system: From patterning to neuronal connectivity. Nat. Rev. Neurosci..

[129] Salinas P.C., Zou Y. (2008). Wnt signaling in neural circuit assembly. Annu. Rev. Neurosci..

[130] Inestrosa N.C., Montecinos-Oliva C., Fuenzalida M. (2012). Wnt signaling: Role in Alzheimer disease and schizophrenia. J. Neuroimmune Pharm..

[131] Hall A.C., Lucas F.R., Salinas P.C. (2000). Axonal remodeling and synaptic differentiation in the cerebellum is regulated by WNT-7a signaling. Cell.

[132] Rosso S.B., Inestrosa N.C. (2013). WNT signaling in neuronal maturation and synaptogenesis. Front. Cell. Neurosci..

[133] Rosso S.B., Sussman D., Wynshaw-Boris A., Salinas P.C. (2005). Wnt signaling through Dishevelled, Rac and JNK regulates dendritic development. Nat. Neurosci..

[134] De Ferrari G.V., Moon R.T. (2006). The ups and downs of Wnt signaling in prevalent neurological disorders. Oncogene.

[135] Alvarez A.R., Godoy J.A., Mullendorff K., Olivares G.H., Bronfman M., Inestrosa N.C. (2004). Wnt-3a overcomes beta-amyloid toxicity in rat hippocampal neurons. Exp. Cell Res..

[136] Inestrosa N., De Ferrari G.V., Garrido J.L., Alvarez A., Olivares G.H., Barria M.I., Bronfman M., Chacon M.A. (2002). Wnt signaling involvement in beta-amyloid-dependent neurodegeneration. Neurochem. Int..

[137] Inestrosa N.C., Toledo E.M. (2008). The role of Wnt signaling in neuronal dysfunction in Alzheimer's Disease. Mol. Neurodegener..

[138] Aghaizu N.D., Jin H., Whiting P.J. (2020). Dysregulated Wnt Signalling in the Alzheimer's Brain. Brain Sci..

[139] Cerpa W., Farias G.G., Godoy J.A., Fuenzalida M., Bonansco C., Inestrosa N.C. (2010). Wnt-5a occludes Abeta oligomer-induced depression of glutamatergic transmission in hippocampal neurons. Mol. Neurodegener..

[140] Vargas J.Y., Fuenzalida M., Inestrosa N.C. (2014). In vivo activation of Wnt signaling pathway enhances cognitive function of adult mice and reverses cognitive deficits in an Alzheimer's disease model. J. Neurosci. Off. J. Soc. Neurosci..

[141] Cisternas P., Zolezzi J.M., Martinez M., Torres V.I., Wong G.W., Inestrosa N.C. (2019). Wnt-induced activation of glucose metabolism mediates the in vivo neuroprotective roles of Wnt signaling in Alzheimer disease. J. Neurochem..

[142] Cerpa W., Gambrill A., Inestrosa N.C., Barria A. (2011). Regulation of NMDA-receptor synaptic transmission by Wnt signaling. J. Neurosci..

[143] Varela-Nallar L., Alfaro I.E., Serrano F.G., Parodi J., Inestrosa N.C. (2010). Wingless-type family member 5A (Wnt-5a) stimulates synaptic differentiation and function of glutamatergic synapses. Proc. Natl. Acad. Sci. USA.

[144] Cerpa W., Godoy J.A., Alfaro I., Farias G.G., Metcalfe M.J., Fuentealba R., Bonansco C., Inestrosa N.C. (2008). Wnt-7a modulates the synaptic vesicle cycle and synaptic transmission in hippocampal neurons. J. Biol. Chem..

[145] Wang Y., Li Y.P., Paulson C., Shao J.Z., Zhang X., Wu M., Chen W. (2014). Wnt and the Wnt signaling pathway in bone development and disease. Front. Biosci..

[146] Lie D.C., Colamarino S.A., Song H.J., Desire L., Mira H., Consiglio A., Lein E.S., Jessberger S., Lansford H., Dearie A.R. (2005). Wnt signalling regulates adult hippocampal neurogenesis. Nature.

[147] Okamoto M., Inoue K., Iwamura H., Terashima K., Soya H., Asashima M., Kuwabara T. (2011). Reduction in paracrine Wnt3 factors during aging causes impaired adult neurogenesis. FASEB J..

[148] Torres V.I., Barrera D.P., Varas-Godoy M., Arancibia D., Inestrosa N.C. (2021). Selective Surface and Intraluminal Localization of Wnt Ligands on Small Extracellular Vesicles Released by HT-22 Hippocampal Neurons. Front. Cell. Dev. Biol..

[149] Godoy J.A., Espinoza-Caicedo J., Inestrosa N.C. (2021). Morphological neurite changes induced by porcupine inhibition are rescued by Wnt ligands. Cel. Commun. Signal.

[150] Bovolenta P., Rodriguez J., Esteve P. (2006). Frizzled/RYK mediated signalling in axon guidance. Development.

[151] Wayman G.A., Impey S., Marks D., Saneyoshi T., Grant W.F., Derkach V., Soderling T.R. (2006). Activity-dependent dendritic arborization mediated by CaM-kinase I activation and enhanced CREB-dependent transcription of Wnt-2. Neuron.

[152] Wayman G.A., Bose D.D., Yang D., Lesiak A., Bruun D., Impey S., Ledoux V., Pessah I.N., Lein P.J. (2012). PCB-95 modulates the calcium-dependent signaling pathway responsible for activity-dependent dendritic growth. Env. Health Perspect..

[153] Yu X., Malenka R.C. (2003). Beta-catenin is critical for dendritic morphogenesis. Nat. Neurosci..

[154] Yu X., Malenka R.C. (2004). Multiple functions for the cadherin/catenin complex during neuronal development. Neuropharmacology.

[155] Peng Y.R., He S., Marie H., Zeng S.Y., Ma J., Tan Z.J., Lee S.Y., Malenka R.C., Yu X. (2009). Coordinated changes in dendritic arborization and synaptic strength during neural circuit development. Neuron.

[156] Sahores M., Salinas P.C. (2011). Activity-mediated synapse formation a role for Wnt-Fz signaling. Curr. Top. Dev. Biol..

[157] Avila M.E., Sepulveda F.J., Burgos C.F., Moraga-Cid G., Parodi J., Moon R.T., Aguayo L.G., Opazo C., De Ferrari G.V. (2010). Canonical Wnt3a modulates intracellular calcium and enhances excitatory neurotransmission in hippocampal neurons. J. Biol. Chem..

[158] Farias G.G., Godoy J.A., Cerpa W., Varela-Nallar L., Inestrosa N.C. (2010). Wnt signaling modulates pre- and postsynaptic maturation: Therapeutic considerations. Dev. Dyn..

[159] Farias G.G., Valles A.S., Colombres M., Godoy J.A., Toledo E.M., Lukas R.J., Barrantes F.J., Inestrosa N.C. (2007). Wnt-7a induces presynaptic colocalization of alpha 7-nicotinic acetylcholine receptors and adenomatous polyposis coli in hippocampal neurons. J. Neurosci. Off. J. Soc. Neurosci..

[160] Varela-Nallar L., Ramirez V.T., Gonzalez-Billault C., Inestrosa N.C. (2012). Frizzled receptors in neurons: From growth cones to the synapse. Cytoskeleton.

[161] Varela-Nallar L., Grabowski C.P., Alfaro I.E., Alvarez A.R., Inestrosa N.C. (2009). Role of the Wnt receptor Frizzled-1 in presynaptic differentiation and function. Neural Dev..

[162] Miech C., Pauer H.U., He X., Schwarz T.L. (2008). Presynaptic local signaling by a canonical wingless pathway regulates development of the Drosophila neuromuscular junction. J. Neurosci. Off. J. Soc. Neurosci..

[163] Lau C.G., Takayasu Y., Rodenas-Ruano A., Paternain A.V., Lerma J., Bennett M.V., Zukin R.S. (2010). SNAP-25 is a target of protein kinase C phosphorylation critical to NMDA receptor trafficking. J. Neurosci. Off. J. Soc. Neurosci..

[164] Munoz F.J., Godoy J.A., Cerpa W., Poblete I.M., Huidobro-Toro J.P., Inestrosa N.C. (2014). Wnt-5a increases NO and modulates NMDA receptor in rat hippocampal neurons. Biochem. Biophys. Res. Commun..

[165] Parodi J., Montecinos-Oliva C., Varas R., Alfaro I.E., Serrano F.G., Varas-Godoy M., Munoz F.J., Cerpa W., Godoy J.A., Inestrosa N.C. (2015). Wnt5a inhibits K^+^ currents in hippocampal synapses through nitric oxide production. Mol. Cell. Neurosci..

[166] McQuate A., Latorre-Esteves E., Barria A. (2017). A Wnt/Calcium Signaling Cascade Regulates Neuronal Excitability and Trafficking of NMDARs. Cell. Rep..

[167] McLeod F., Bossio A., Marzo A., Ciani L., Sibilla S., Hannan S., Wilson G.A., Palomer E., Smart T.G., Gibb A. (2018). Wnt Signaling Mediates LTP-Dependent Spine Plasticity and AMPAR Localization through Frizzled-7 Receptors. Cell. Rep..

[168] Ramos-Fernandez E., Tapia-Rojas C., Ramirez V.T., Inestrosa N.C. (2019). Wnt-7a Stimulates Dendritic Spine Morphogenesis and PSD-95 Expression Through Canonical Signaling. Mol. Neurobiol..

[169] Ramos-Fernandez E., Arrazola M.S., Oliva C.A., Arredondo S.B., Varela-Nallar L., Inestrosa N.C. (2021). Wnt5a promotes hippocampal postsynaptic development and GluN2B-induced expression via the eIF2alpha HRI kinase. Sci. Rep..

[170] Martinez M., Torres V.I., Vio C.P., Inestrosa N.C. (2020). Canonical Wnt Signaling Modulates the Expression of Pre- and Postsynaptic Components in Different Temporal Patterns. Mol. Neurobiol..

[171] Cabeza C., Figueroa A., Lazo O.M., Galleguillos C., Pissani C., Klein A., Gonzalez-Billault C., Inestrosa N.C., Alvarez A.R., Zanlungo S. (2012). Cholinergic abnormalities, endosomal alterations and up-regulation of nerve growth factor signaling in Niemann-Pick type C disease. Mol. Neurodegener..

[172] Cerpa W., Ramos-Fernandez E., Inestrosa N.C. (2014). Modulation of the NMDA Receptor Through Secreted Soluble Factors. Mol. Neurobiol..

[173] Saarikangas J., Kourdougli N., Senju Y., Chazal G., Segerstrale M., Minkeviciene R., Kuurne J., Mattila P.K., Garrett L., Holter S.M. (2015). MIM-Induced Membrane Bending Promotes Dendritic Spine Initiation. Dev. Cell..

[174] Alvarez V.A., Sabatini B.L. (2007). Anatomical and physiological plasticity of dendritic spines. Annu. Rev. Neurosci..

[175] Luikart B.W., Zhang W., Wayman G.A., Kwon C.H., Westbrook G.L., Parada L.F. (2008). Neurotrophin-dependent dendritic filopodial motility: A convergence on PI3K signaling. J. Neurosci. Off. J. Soc. Neurosci..

[176] Wolf A.M., Lyuksyutova A.I., Fenstermaker A.G., Shafer B., Lo C.G., Zou Y. (2008). Phosphatidylinositol-3-kinase-atypical protein kinase C signaling is required for Wnt attraction and anterior-posterior axon guidance. J. Neurosci. Off. J. Soc. Neurosci..

[177] Pan W., Choi S.C., Wang H., Qin Y., Volpicelli-Daley L., Swan L., Lucast L., Khoo C., Zhang X., Li L. (2008). Wnt3a-mediated formation of phosphatidylinositol 4,5-bisphosphate regulates LRP6 phosphorylation. Science.

[178] Slusarski D.C., Corces V.G., Moon R.T. (1997). Interaction of Wnt and a Frizzled homologue triggers G-protein-linked phosphatidylinositol signalling. Nature.

[179] Lalo U., Nezis I.P., Pankratov Y. (2022). Impact of Autophagy Impairment on Experience- and Diet-Related Synaptic Plasticity. Int. J. Mol. Sci..

[180] Rios J.A., Godoy J.A., Inestrosa N.C. (2018). Wnt3a ligand facilitates autophagy in hippocampal neurons by modulating a novel GSK-3beta-AMPK axis. Cell. Commun. Signal..

[181] Koval A., Katanaev V.L. (2011). Wnt3a stimulation elicits G-protein-coupled receptor properties of mammalian Frizzled proteins. Biochem. J..

[182] Ramirez V.T., Ramos-Fernandez E., Henriquez J.P., Lorenzo A., Inestrosa N.C. (2016). Wnt-5a/Frizzled9 Receptor Signaling through the Galphao-Gbetagamma Complex Regulates Dendritic Spine Formation. J. Biol. Chem..

[183] Ramirez V.T., Ramos-Fernandez E., Inestrosa N.C. (2016). The Galphao Activator Mastoparan-7 Promotes Dendritic Spine Formation in Hippocampal Neurons. Neural Plast..

[184] Budnik V., Salinas P.C. (2011). Wnt signaling during synaptic development and plasticity. Curr. Opin. Neurobiol..

